# Extracellular Vesicles and Cell Pathways Involved in Cancer Chemoresistance

**DOI:** 10.3390/life12050618

**Published:** 2022-04-21

**Authors:** Lara Console, Mariafrancesca Scalise

**Affiliations:** Department of Biology, Ecology and Earth Sciences (DiBEST), Laboratory of Biochemistry, Molecular Biotechnology and Molecular Biology, University of Calabria, Via P. Bucci 4c, 87036 Arcavacata di Rende, Italy

**Keywords:** exosomes, cancer, membrane transporters, SLC, ABC, chemoresistance, drugs

## Abstract

Chemoresistance is a pharmacological condition that allows transformed cells to maintain their proliferative phenotype in the presence of administered anticancer drugs. Recently, extracellular vesicles, including exosomes, have been identified as additional players responsible for the chemoresistance of cancer cells. These are nanovesicles that are released by almost all cell types in both physiological and pathological conditions and contain proteins and nucleic acids as molecular cargo. Extracellular vesicles released in the bloodstream reach recipient cells and confer them novel metabolic properties. Exosomes can foster chemoresistance by promoting prosurvival and antiapoptotic pathways, affecting cancer stem cells and immunotherapies, and stimulating drug efflux. In this context, a crucial role is played by membrane transporters belonging to ABC, SLC, and P-type pump families. These proteins are fundamental in cell metabolism and drug transport in either physiological or pathological conditions. In this review, different roles of extracellular vesicles in drug resistance of cancer cells will be explored.

## 1. Introduction

Cancer is the second leading cause of death globally, representing one of the most significant public health problems [1]. Although novel therapeutic strategies have been developed, chemotherapy remains the leading approach for tumor treatment [2]. Despite significant advances in increasing the efficacy of chemotherapeutics, chemoresistance remains one of the major obstacles in cancer treatment and represents a significant risk to the survival of patients [3,4,5]. Two main classes of chemoresistance can be distinguished: intrinsic and acquired [6]. The intrinsic resistance is associated with factors, such as stem cells in the tumor mass, present before any drug exposure. The acquired resistance is a stepwise process involving various mechanisms, including increased DNA repair, altered expression of oncogenes or tumor suppressor genes, altered drug targets, increased drug efflux, and increased autophagy [7,8]. A growing body of evidence suggests that extracellular vesicles can play a role in mediating drug resistance of cancer cells [9]. Indeed, these vesicles, released from almost all cells, carry different cargo types, including mRNA, microRNA, long noncoding RNA, lipids, and proteins, that may influence the phenotype and metabolisms of recipient cells even they are located far from the donors [7,10]. 

**Figure 1:**
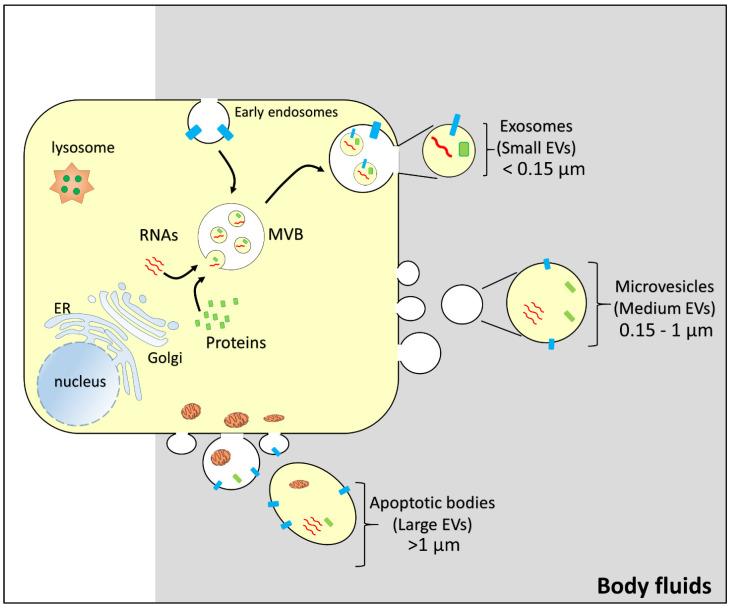
Representation of the biogenesis of vesicles. From a generic donor cell, budding of different vesicles: exosomes/small extracellular vesicles (size: less than 0.15 µm); microvesicles/medium extracellular vesicles (size: 0.15–1 µm); apoptotic bodies/large extracellular vesicles (size: greater than 1 µm). MVB, multivesicular body; EV, extracellular vesicle. Intracellular organelles, RNAs (red), and proteins (green and light blue) are indicated.

The cargo of extracellular vesicles changes accordingly with the physio-pathological conditions of the donor cells [10,11]. In the chemoresistance scenario, extracellular vesicles from drug-resistant cancer cells can provide drug-sensitive cells with nucleic acids and proteins that confer a resistance phenotype (Figure 1 and Table 1). Moreover, some evidence demonstrates that extracellular vesicles can be directly involved in drug extrusion from cancer cells [12]. The understanding of vesicle-mediated drug resistance is required for finding new targets of chemoresistance, as well as to identify suitable biomarkers devoted to managing treatments at the earliest sign of drug resistance. Since vesicles can be collected from body fluids such as blood, urine, and saliva, they represent an easily accessible source of biomarkers for monitoring the effectiveness of therapy [10]. Besides other advantages and novelties, detecting biomarkers from body fluids via noninvasive methods also avoids the harm and discomfort of solid tissue biopsy [13].

Extracellular vesicles (EVs) can be classified into different subtypes. Using the nomenclature suggested by Minimal Information for Studies of Extracellular Vesicles 2018 (MISEV2018) [69], it is possible to distinguish three different subtypes of vesicles: large EVs (>1 µm), medium EVs (>150 nm and <1 µm), and small EVs (<150 nm) [69,70]. Another possible classification is based on the cell origin of the extracellular vesicles. A first class includes apoptotic bodies, which bud directly from the plasma membrane during late apoptosis and contain disaggregated cell components and organelles. The medium and small size extracellular vesicles are also, respectively, described as microvesicles that directly bud from the cell membrane and exosomes which originate from the exocytic fusion of multivesicular bodies with the plasma membrane (Figure 1).

However, due to the heterogeneity in terms of size and the overlap of some biochemical characteristics (markers) between exosomes and microvesicles, it is difficult to unequivocally distinguish these two subgroups of vesicles [71,72]. As a consequence, most studies on the extracellular vesicles are carried out on a mixture of exosomes and microvesicles. For this reason, we will use the term EVs instead of exosomes or microvesicles throughout the review.

Another important issue to consider about extracellular vesicles is the possible coisolation of particles such as ribonucleoprotein aggregates, lipoproteins, and exomers [69]. For instance, miRNAs bound with high-density lipoproteins may be co-isolated as a contaminant of miRNAs embedded into extracellular vesicles [73]. Thus, contaminants can affect the correct interpretation of the experimental results. Further analyses of the isolated EVs to determine the presence and the proportion of such contaminants are highly desirable [69]. However, in certain cases such as when only small amounts of working materials are available, these additional investigations cannot be performed. In this case, as suggested by MISEV2018, the results will be examined as action mediated by EV-enriched preparations, rather than EV-specific activity [69].

## 2. Extracellular Vesicles, Drug Efflux, and Membrane Transporters

Drug efflux is a common mechanism underlying the chemoresistance of cancer cells. In this respect, vesicles have been shown to play some key roles. One of the most common cancer features is the presence of an acidic microenvironment primarily due to altered glycolysis and hypoxia that causes the production of lactic acid and release of protons to the extracellular milieu [74,75]. Recent studies demonstrated a link between microenvironment acidity and increased secretion of vesicles [76,77]. In particular, there is evidence that drug-resistant cancer cells secrete more exosomes than sensitive cells and that these vesicles contain anticancer drugs. Accordingly, Federici et al. showed that human melanoma cells could develop resistance against cisplatin, exporting the drug via vesicles whose release is increased in the presence of an acidic environment [78]. The pretreatment of melanoma cells with the proton pump inhibitor lansoprazole reduces exosome secretion and improves cisplatin efficacy [78]. Accordingly, in vitro and in vivo experiments showed that inhibition of vesicle biogenesis enhances the accumulation of doxorubicin and pixantrone in B-cell lymphoma [79]. The treatment of breast and ovarian cancer cells with doxorubicin and pixantrone leads to increased secretion of vesicles containing considerable amounts of these drugs, compared to untreated cells that in turn induce resistance [80].

Another mechanism by which vesicle secretion can be increased is based on annexin A3 action. Upregulation of annexin A3, belonging to the phospholipid-binding protein family, induces biogenesis and release of exosomes [81]. According to that, it was found that this protein is enriched in drug-resistant ovarian cancer cells and its upregulation correlated with the reduction in the intracellular Pt-drug concentration, which in turn prevents apoptosis [7,82]. Moreover, annexin A3 was also directly found in extracellular vesicles, suggesting that the transfer of this protein may induce chemoresistance in recipient cells [81].

Altogether, these findings correlate with a well-assessed role of vesicles in removing toxic compounds from cells, representing a first biological function ascribed to exosome secretion.

In the context of vesicle-mediated drug resistance caused by the efflux or improper accumulation of therapeutic agents in cancer cells, membrane transporters can be involved as primary players.

Membrane transporters are classified into two major groups, according to the source of energy used for the transport reaction, namely ABC and SLC super families. ATP-binding cassettes (ABCs) are primary active transporters since they use ATP hydrolysis as energy for driving transport [83,84], whereas solute carriers (SLCs) are secondary active transporters that exploit the gradients of ions or other substrates across the cell membranes [85,86]. Another class of proteins, called P-type pumps, involved in many cell processes and present in virtually all living organisms, have to be added to the list of transporters [87]. These proteins share with the ABCs the source of energy, namely ATP hydrolysis.

The following sections will deal with the link between exosome-mediated drug resistance and transporters belonging to ABC, SLC, and P-type pump families.

### 2.1. ABC Transporters and Exosomes in Drug Efflux

ABCs originated early during evolution and are conserved across all kingdoms of life, sharing some structural features: regardless of being prokaryotic or eukaryotic proteins, ABCs are constituted by two transmembrane domains (TMDs) and two nucleotide-binding domains (NBDs) which are involved in the binding and hydrolysis of ATP [88,89]. ABC are classified into seven families, including more than 50 members in humans [88] (https://www.genenames.org/data/genegroup/#!/group/417, accessed on 19 March 2022). An eminent example of ABC transporters involved in drug efflux is given by the P-glycoprotein (P-gp), also known as ABCB1 or MDR1 [90,91,92]. Among the ABC transporters, P-gp is the best studied due to the discovery of its role in cancer development and progression [90,91,92]. Indeed, P-gp is famous as a drug transporter mainly involved in the efflux of chemotherapy agents and is, hence, one of the most relevant players in chemoresistance. Notwithstanding the large variety and different structures, a common feature among the substrates recognized by P-gp is the hydrophobic nature of the molecules [90,91,92]. This is probably due to the peculiar transport mechanism of P-gp that consists of anchoring the substrate to the membrane before the actual translocation through P-gp, coupled to the hydrolysis of ATP [93].

Given the role of P-gp in mediating drug efflux, strong efforts have been made to identify good and specific inhibitors [94]. However, an intriguing paradox exists: indeed, a pharmacological molecule, even though tested as an inhibitor, may behave as a substrate of P-gp. Indeed, a sizable number of drugs failed to be true inhibitors of P-gp, being rather substrates of the protein [94]. These results made it difficult to overcome the chemoresistance linked to P-gp overexpression in human cancers.

This scenario is made even more complex considering that several SNPs have been identified and annotated in P-gp, further influencing the substrate specificity [95]. Moving from these premises, it is not surprising that alteration of the P-gp expression profoundly influences the disposition and pharmacodynamics of many anticancer drugs [96].

Several studies reported the presence of P-gp in extracellular vesicles that can induce chemoresistance in sensitive recipient cancer cells [53,62,97]. In particular, P-gp is inserted in the membrane of vesicles as in the native membrane, i.e., with the transmembrane-spanning domain crossing the vesicle bilayer [97]. For instance, vesicles derived from docetaxel-resistant prostate cancer cells (PC3) transfer the drug resistance to sensitive cells by delivering P-gp as a cargo [62]. Similarly, extracellular vesicles released from doxorubicin-resistant osteosarcoma cells induce resistance in recipient cells by delivering P-gp mRNA and protein [53] (Figure 1 and Table 2). Another study demonstrated that the P-gp level in blood vesicles from patients with docetaxel-resistant prostate cancer was relatively higher than in patients that did not receive therapy [62].

The transient receptor potential channel 5 (TrpC5) was also found in extracellular vesicles deriving from adriamycin-resistant Michigan Cancer Foundation (MCF)-7 cells (Table 2) [56,98]. The transfer of TrpC5 through the vesicular membrane confers the phenotype of resistance in drug-sensitive cells. The acquisition of exosomal TrpC5 leads to Ca^2+^ influx in breast cancer recipient cells. The increase in Ca^2+^ influx was specifically ascribed to TrpC5, as confirmed by using specific TrpC5 inhibitors as well as blockers of other cell Ca^2+^ channels. The increase in intracellular Ca^2+^ concentration of recipient cells caused the nuclear translocation of the transcription factor Nuclear Factor of Activated T-cells Cytoplasmic 3 (NFATC3), which induces P-gp overexpression [56]. Similarly, the adriamycin-resistant breast cancer cells secreted vesicles containing UCH-L1 (Table 2) [54]. It is a member of the ubiquitin carboxyterminal hydrolase (UCH) family, which upregulates P-gp expression through the MAPK/ERK signaling pathway [105]. The uptake of UCH-L1-containing vesicles into adriamycin-sensitive breast cancer cells transferred the chemoresistance phenotype [54]. Furthermore, it has been reported that vesicles released by docetaxel-resistant breast cancer cell line (MCF-7) rendered recipient-sensitive MCF-7 cells resistant to the same drug by transferring the P-gp protein [55].

**Figure 2:**
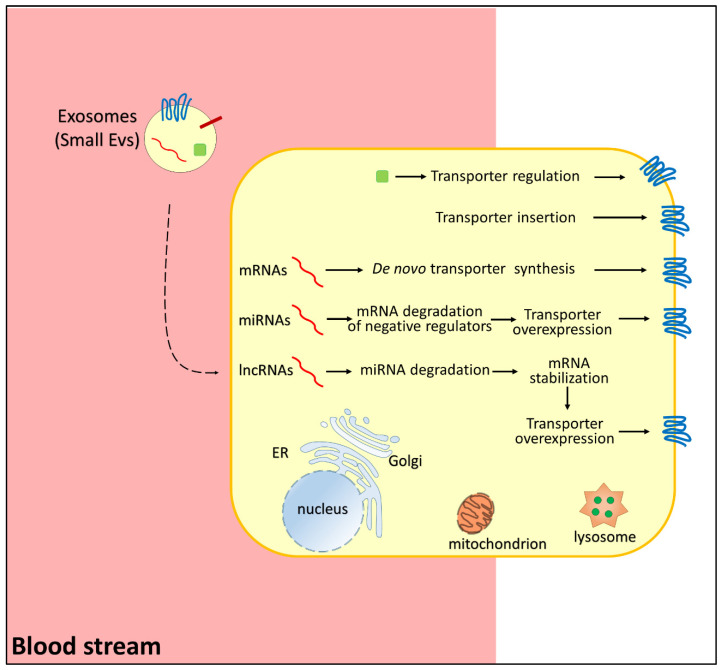
Representation of pathways regulating membrane transporters in chemoresistance. Small extracellular vesicles carry RNAs (in red, mRNA, miRNA, lncRNA), regulatory proteins (green), and membrane transporters (blue). In the donor cells, mRNAs induce de novo transporter synthesis, miRNAs reduce the expression of negative regulators of transporter function/expression, lncRNAs induce miRNA degradation with consequent transporter overexpression, proteins (green) regulate transporter function, and transporter(s) carried by small EVs are inserted directly in the membrane.

Besides proteins, extracellular vesicles can deliver other bioactive molecules, such as nucleic acids (Figure 2 and Table 2). As an example, extracellular vesicles derived from cancer-associated fibroblasts (CAFs) carry a specific long noncoding RNA (lncRNA), named LINC00355. This lncRNA is responsible for buffering the miRNA miR-34b-5p, known as a modulator of the P-gp protein [47]. The absorption of CAF vesicles, carrying LINC00355, by bladder cancer cells triggers the overexpression of P-gp with the consequent acquirement of a cisplatin-resistance phenotype (Table 2) [47].

A mechanism based on miRNA has also been described for ovarian cancer; in this case, the resistance of cancer cells to paclitaxel is mediated by exosomes containing miR-1246, which can target caveolin 1, i.e., a regulator of P-gp expression (Table 2). Therefore, miR-1246 released into recipient cancer cells by extracellular vesicles reduces the expression of caveolin 1, which triggers the overexpression of P-gp1, causing resistance to paclitaxel [34].

Another protein involved in drug resistance is multidrug resistance protein 1 (MRP1), also known as ABCC1 [106]. MRP1 is mainly localized at the basolateral surface of epithelial and the apical surface of brain capillaries [107]. This localization allows the efflux towards the blood of the MRP1 substrates contributing to drug and xenobiotic disposition in normal and cancer cells [106,107]. Like P-gp, ABCC1 recognizes a large variety of antineoplastic drugs, antivirals, and toxicants [106,107]. Interestingly, vesicles released from non-small-cell lung cancer contain the circular RNA phosphatidylinositol-4-phosphate 5-kinase type 1 alpha (circ_PIP5K1A) [49]. This molecule has miR-101 as a primary target; the disruption of miR-101 causes the overexpression of ABCC1 given that ABCC1 protein levels are negatively regulated by miR-101 [49]. Therefore, the miR-101/ABCC1 axis is disrupted in recipient cells, and these acquire a cisplatin-resistant phenotype (Table 2). Conversely, the knockdown of exosomal circ_PIP5K1A promoted cisplatin sensitivity in recipient lung cancer cells [49].

Another member of the ABC superfamily with a well-acknowledged role in cancer resistance is ABCG2, better known as breast cancer resistance protein (BCRP). As indicated by the name, this protein was first identified in breast cancer cells (MCF-7) as highly resistant to doxorubicin. Then, it was discovered that this protein is mainly expressed in the placental syncytiotrophoblasts, in the canalicular membrane of the hepatocytes, and on the luminal side of epithelial cells in the small and large intestine [108]. ABCG2 recognizes as a substrate a broad spectrum of anticancer drugs, sulfate and glucuronide conjugates of xenobiotics, natural compounds, and toxins [108]. In the frame of chemoresistance related to extracellular vesicles, some reports indicated regulation of ABCG2 expression by long noncoding RNAs and circular RNA present in exosomes released by cancer cells (Figure 2 and Table 2). For example, the long noncoding RNA known as linc-VLDLR is an upstream positive regulator of ABCG2 [40]. The linc-VLDLR is highly present in hepatocellular cancer (HCC) cells resistant to sorafenib, camptothecin, and doxorubicin [99]. Interestingly, vesicles released by the resistant cells contain linc-VLDLR as a cargo. The exposure of sensitive HCC cells to extracellular vesicles carrying linc-VLDLR increased ABCG2 expression, with a consequent reduction in cancer cell death [99]. The same mechanism has also been described for the linc-VLDLR/ABCG2 axis in esophageal cancer cells [40].

Finally, another link between extracellular vesicles, ABC transporters, and chemoresistance has been proposed: indeed, besides other mechanisms, the biogenesis of vesicles in hematological neoplasm is dependent on the lipid transporter ABCA3 (Table 2). ABCA3 levels in malignant lymphoma and myeloma are augmented with a consequent increase in exosome secretions. This phenomenon is linked to the resistance to anticancer drugs such as adriamycin, picoxenone [109], and rituximab [67].

### 2.2. P-Type ATPases and Extracellular Vesicles

The P-type ATPase family is formed by five groups (indicated by I–V symbols), each including different classes able to recognize a specific ion as a substrate [87]. These proteins are constituted by an even number of membrane-spanning domains with molecular masses ranging from 70 to 150 kDa [87]. As suggested by the name, P-type ATPases can hydrolyze ATP, allowing for ion translocation through the cell membranes [87]. The overall architecture of these proteins is well conserved within the family members and consists of four domains indicated as P-domain, N-domain, A-domain, and M-domain [87]. The P-domain is the phosphorylation site, being the core of the catalytic mechanism, and includes the N-domain, i.e., the ATP-binding domain. The A-domain is located at the N-terminus of the pumps and is responsible for the conformational changes occurring during the transport cycle; it is plausible that the A-domain plays a regulatory role [87]. Finally, the M-domain is the transmembrane portion of the pump responsible for the ion-path formation [87]. Two P-type pumps have been identified as involved in the resistance to Pt-based drugs: the copper efflux transporters ATP7A and ATP7B (Table 2) [61]. These participate in heavy metal detoxification, thus being crucial for cell life [61,110]. Therefore, it is not surprising that alterations of human ATP7A and ATP7B are associated with Wilson’s disease, which is characterized by abnormal copper accumulation in the liver and the brain [110]. In contrast to Wilson’s phenotype, the upregulation of ATP7A and ATP7B has been associated with Alzheimer’s disease and chemoresistance. Interestingly, the authors of [111] found that vesicles released by ovarian cancer cells resistant to Pt-based drugs harbor higher amounts of ATP7A and ATP7B than exosomes derived from drug-sensitive cells. The same authors also described augmented exosome secretion from resistant cells compared to that from their sensitive counterparts [61].

### 2.3. Solute Carrier (SLC) Transporters and Extracellular Vesicles

SLC transporters are classified into 60 families, including more than 500 members. In humans, indeed, SLCs are involved in the absorption of several nutrients and cofactors and their distribution in intracellular organelles [112]. Moreover, SLCs are responsible for the excretion of catabolites and reabsorption phenomena occurring in the kidney [112]. The role of SLCs in mediating cancer chemoresistance is well acknowledged due to their ability to mediate drug transport, besides their physiological substrates [113,114,115,116]. Several SLCs can be found in the dedicated database of exosome cargos, named Exocarta [117], which also collects the rough proteomic data about exosomes released by cancer cells; however, only little information is currently available on the function of these proteins as exosome cargo(s) [35,100,101,118].

However, some information that links SLCs to drug resistance induced by extracellular vesicles is available (Figure 2). An eminent example is the case of the equilibrative nucleoside transporter 2 (ENT2) [35,119]. It belongs to the SLC29 family and is responsible for the uptake of purine and pyrimidine nucleosides [120]. ENT2 is a ubiquitous protein mainly localized at the plasma membrane but has also been detected in nuclear membranes. Besides nucleobases, ENT2 is involved in the transport of nucleoside-based drugs [120]. The ability to recognize several pharmacological compounds as substrates made this protein a hot pharmacological target for several diseases, including cancer and acquired immunodeficiency syndrome (AIDS) [121]. In the frame of chemoresistance mediated by extracellular vesicles, the role of ENT2 has been linked to exosomes released from cancer-associated fibroblasts (CAFs) to lymphoma [35,119]. Indeed, CAFs support lymphoma cell growth and chemoresistance to gemcitabine and cytarabine by downregulating ENT2 expression (Figure 1) [122]. CAF-derived vesicles were found to contain the miRNA-4717-5p that targets a deubiquitinase, triggering a cascade event: the disruption of deubiquitinase causes higher ubiquitination of ENT2 with following faster ENT2 degradation (Table 2). Ultimately, increased resistance to anticancer drugs is observed [35].

Another SLC member involved in chemoresistance is SL9A1, known as NHE1 [123]. This protein belongs to the SLC9 family, a subgroup of the eukaryotic and prokaryotic monovalent cation proton antiporter (CPA) superfamily (Transport Protein Database http://tcdb.ucsd.edu/tcdb/, accessed on 19 March 2022) [124,125]. NHE1 is a glycosylated plasma membrane protein with a large hydrophilic C-terminus responsible for regulating its activity [126]. NHE1 is ubiquitously expressed in mammalian cells and is responsible for the exchange of Na^+^ and H^+^ with the consequent alkalization of the cell in defense of H^+^ derived from metabolism or electrically driven H^+^-accumulation [124,125]. NHE1 plays a crucial role in cell migration, proliferation, and death, and it has been linked to the sensitivity of breast cancer cells to cisplatin [125]. In good agreement, silencing of SCL9A1 was responsible for the inhibition of cell migration, inhibition, and matrix metalloproteinase production in breast cancer cells [127].

Interestingly, extracellular vesicles derived from adipose mesenchymal stem cells (AdMSC-Exos) were shown to have an antiproliferative effect in recipient breast cancer cells, ameliorating their drug resistance [100].

Indeed, these vesicles carry the miRNA miR-1236, which is an upstream negative regulator of NHE1 in breast cancer cells where a link between NHE and the Wingless-related integration site (Wnt)–catenin pathway has been proposed [100]. NHE1 activates β-catenin which, in turn, induces cancer cell proliferation and resistance to DPP. Therefore, when vesicles containing miR-1236 enter breast cancer cells, the degradation of NHE1 mRNA is responsible for the above-described antiproliferative effects [100].

Another SLC protein linked to exosomes and chemoresistance is SLC7A11, known as xCT [128]. This protein forms a functional heterodimer with the glycoprotein CD98 (SLC3A2) via a disulfide bond between two conserved cysteine residues, whose 3D structure has been recently determined by CryoEM [129]. The protein xCT is well acknowledged as a crucial player in cancer development and progression [130], being overexpressed in virtually all human cancers, despite the narrower expression in nonpathological conditions [131]. xCT is a plasma membrane transporter responsible for exchanging cystine with glutamate with a 1:1 stoichiometry; the cystine taken up from the extracellular milieu is reduced to cysteine for GSH synthesis [132]. This transport mechanism underlies the role of xCT in the redox homeostasis of cells and, then, in the oxidative stress response. Indeed, xCT has been described as the mediator of a recently discovered cell death pathway called ferroptosis, characterized by an iron-dependent abnormal peroxidation [133]. In particular, xCT impairment causes cystine depletion and consequent accumulation of lipid hydroperoxides [134]. The involvement in human cancers prompted the research of drugs with inhibitory effects on the xCT transport activity, even though a link with chemoresistance and poor survival of glioblastoma patients has been proposed [135].

In the context of anticancer research, an inhibitor of xCT that has been approved by FDA is sulfasalazine (SAS). SAS upregulates ROS via xCT inhibition leading to cell death [136]. In the exosome scenario, a link between xCT and immune checkpoint blockade (ICB) therapy has been proposed in melanoma patients [101]. The study proposed that the inhibition of xCT by SAS is strategic for treating melanoma patients in line with the altered levels of glutamate found in cancer cells [101]. However, in the same study, it has been shown that extracellular vesicles, released from melanoma cells treated with SAS, reduced the efficacy of the ICB therapy [101]. Indeed, ICB therapy aims at enhancing antitumoral immunity by acting on specific immune system components [137]. Therefore, the authors concluded that melanoma patients affected by inflammatory diseases may not be eligible for the combination of SAS and immunotherapy [101].

## 3. Extracellular-Vesicle-Induced Drug Resistance Promoting Prosurvival and Antiapoptotic Pathways

**Figure 3:**
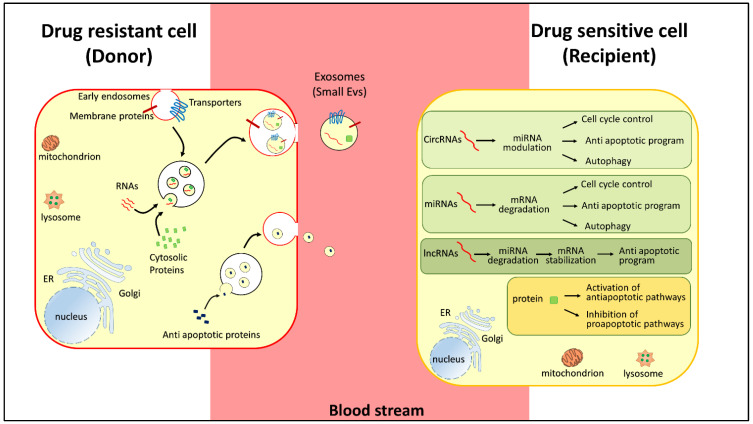
Representation of pathways regulating chemoresistance. Small extracellular vesicles carry RNAs (in red, mRNA, miRNA, lncRNA), regulatory proteins (green), and membrane transporters (blue) released by chemoresistant cancer cells (donor-red membrane). In the donor cells, RNAs (in red, circRNAs, miRNAs, and lncRNAs) and proteins (green) regulate cell cycle control, proapototic and antiapoptic programs, and autophagy. The donor cells release antiapoptotic proteins (blue) to activate prosurvival pathways.

A growing body of evidence has demonstrated that exosomes can mediate drug resistance by modulating several cell processes (Figure 3) [3,7,138]. One of these is apoptosis, which is disturbed in both acquired and intrinsic resistance to chemotherapies [7,138]. Indeed, drug resistance frequently makes tumor cells able to inhibit apoptosis, resulting in cancer cell survival and unfavorable outcomes [139]. Extracellular vesicles can decrease proapoptotic signaling in the donor cells or increase antiapoptotic signaling in the recipient cells (Figure 3 and Table 2) [140].

In the first case, vesicles embed and remove proapoptotic proteins such as caspase-3 from the donor cells. The decrease in the intracellular concentration of caspase-3 promotes the shift towards an antiapoptotic state of the donor cells [141]. As demonstrated by Boing et al., inhibition of the release of vesicles carrying caspase-3 rescued the normal level of apoptosis in endothelial donor cells [141]. Another example of this mechanism occurs in colorectal cancer, where the tumor-suppressive miRNAs miR-145 and miR-34 are removed from cells through extracellular vesicles. This triggers a decrease in apoptosis level and an increase in 5-fluorouracil resistance of these cells (Table 2) [30].

In recipient cells, extracellular vesicles can promote tumor survival by supplying nucleic acids and proteins that directly interfere with antiapoptotic pathways [39,43] (Figure 3). As an example, vesicles released by platelets carry the membrane receptor CD41 (integrin a-IIb), which interacts with the extracellular matrix of cancer cells such as bone marrow myeloma cells [140]. The interaction with CD41-related exosomes reduces the phosphorylation of the c-Jun-N terminal kinase (JNK), with a consequent increase in antiapoptotic proteins such as Bcl-2 and reduced cleavage of caspase-3 [102]. For instance, vesicles can promote cell survival by delivering the lncRNA small nucleolar RNA host gene 14 (SNHG14) to recipient cells [43]. Indeed, the upregulation of SNHG14 has been associated with trastuzumab-resistance of HER2^+^ breast cell lines by targeting the apoptosis regulator Bcl-2/Bax signaling pathway. This lncRNA is highly expressed in vesicles derived from resistant cells [43]. The incubation of sensitive cells with vesicles containing SNHG14 induces trastuzumab resistance (Table 2) [43]. Following that, higher vesicular levels of lncRNA SNHG14 were detected in the serum of patients who exhibited resistance compared to responsive patients [43].

The Bcl-2/Bax pathway was also found to be modulated by another lncRNA named PART1, secreted into exosomes that disseminate drug resistance to sensitive cells (Figure 1 and Table 2) [39]. Indeed, in esophageal squamous cell carcinoma, PART1 promotes resistance to gefitinib because it is able to competitively bind miR-129 which in turn facilitates the expression of the antiapoptotic Bcl-2 protein [39] (Figure 3 and Table 2). Moreover, extracellular PART1, embedded into vesicles, can transfer the gefitinib resistance to recipient cells with the same molecular mechanism. In good agreement, high serum levels of PART1 were associated with poor response to patient treatment [39].

Furthermore, vesicles can confer resistance by delivering miRNAs into chemosensitive tumor cells, altering cell cycle control and inducing antiapoptotic programs (Figure 3) [23]. This is the case of vesicular miR-21 that induced cisplatin resistance in oral squamous cell carcinoma by targeting phosphatase and tensin homolog (PTEN) and programmed cell death protein 4 (PDCD4), which are tumor suppressors involved in apoptosis, cell transformation, invasion, and tumor progression [27]. PTEN is also suppressed through the phosphatidylinositol 3-kinase/protein kinase B (PI3K/Akt) pathway activation induced by vesicular miR-32-5p (Table 2). Indeed, in hepatocellular carcinoma, drug-resistant cells deliver miR-32-5p-containing exosomes inducing resistance into sensitive counterparts by modulating angiogenesis and epithelial–mesenchymal transition [29]. miRNAs also target TP53 [142], another crucial player in cell cycle arrest and apoptosis [36]. Alterations of its expression and function cause resistance to canonical anticancer drugs. In prostate cancer cells, vesicles induce resistance to cisplatin, docetaxel, and doxorubicin through the action of miR-27a, which targets p53 mRNA (Table 2) [36].

Another cell process enhanced in resistant cancer cells is autophagy [143]. The activation of autophagy in response to environmental stress, including that deriving from chemotherapeutic agents, helps cell survival and is implicated in the development of drug resistance [144]. For instance, vesicles derived from hepatitis B virus (HBV)-associated liver cancer cells induce oxaliplatin resistance by activating chaperone-mediated autophagy [103]. In the case of non-small-cell lung cancer, vesicles can mediate chemoresistance by delivering miR-425-3p, whose transcription is upregulated by cisplatin-induced c-Myc. Vesicular miR-425-3p confers chemoresistance through the activation of autophagy targeting AKT1 [14] (Table 2). Moreover, glioblastoma-derived stem cells secrete exosomes that enhance chemoresistance; indeed, these vesicles contain programmed death-ligand 1 (PD-L1), which activates the AMP-activated protein kinase (AMPK)/Unc-51-like kinase 1 (ULK1) pathway mediating autophagy activation that, in turn, results in the increased TMZ-resistance in glioblastoma cells (Table 2) [68]. Vesicular circulating plasmacytoma variant translocation 1 (PVT1) was found to facilitate drug resistance in gastric cancer cells; it induces autophagy activation by modulating the miR-30a-5p, whose target is YAP1, a factor involved in the transcriptional modulation of several genes related to cell proliferation and apoptosis suppression [50].

Finally, vesicles can promote tumor cell survival by triggering DNA repair [145]. Indeed, many anticancer agents target cancer cells, inducing DNA lesions [146,147]. Vesicles that contain in their lumen a long noncoding RNA named SBF2 antisense RNA1 (lncRNA SBF2-AS1) are secreted by glioblastoma cells resistant to temozolomide (Table 2). SBF2-AS1 acts on miR-151a that normally represses X-ray repair cross-complementing 4 (XRCC4) [42,148]. Consequently, vesicles from temozolomide-resistant glioblastoma cells spread a resistant phenotype, delivering high levels of SBF2-AS1 to sensitive cells, which in turn deregulates XRCC4 and enhances the DNA double-strand break repair process [42,148]. Vesicles from reactive astrocytes spread temozolomide resistance by delivering O6-alkylguanine DNA alkyltransferase (MGMT) mRNA to glioma cells. Indeed, MGMT plays a crucial role in repairing DNA damage induced by temozolomide [51].

## 4. Cancer Stem Cell Derived Vesicles and Chemoresistance

Tumors consist of a heterogeneous population of stromal cells, immune cells, fibroblasts, and cancer stem cells (CSCs) [149]. These are characterized by self-renewal capacity, upregulation of drug efflux pumps, increased DNA repair, and dormancy [150]. As a consequence of the above-mentioned features, CSCs are resistant to standard therapies such as anticancer drug treatments and radiotherapy [151]. Several studies individuate extracellular vesicles as the carrier through which CSCs transfer active molecules to non-CSCs for drug resistance. It was demonstrated that CSCs isolated from gemcitabine-resistant pancreatic cancer release vesicles that can stimulate the expression of drug-resistance-related proteins, such as P-gp, Y-box binding protein 1, and breast cancer resistance protein, in a drug-sensitive pancreatic cancer cell line [104]. Moreover, these vesicles are enriched in miR-210 [104], which can inhibit drug-induced apoptosis and increase the phosphorylation of ribosomal protein S6 kinase beta-1, a downstream target of mTOR [152]. Another miRNA associated with chemoresistant phenotype is miR-92a-3p (Table 2).

miR-92a-3p, embedded into vesicles secreted by cancer-associated fibroblasts, induces drug resistance in colorectal cancer cells, promoting cell stemness phenotype and epithelial–mesenchymal transition (EMT) [31]. Indeed, miR-92a-3p targets F-box and WD repeat domain-containing 7 (FBXW7) and modulator of apoptosis protein 1 (MOAP1), which are involved in the modulation of mTOR and apoptosis, respectively (Table 2) [31,153]. Cancer-associated fibroblasts exosomes can also transfer IL-6, activin-A, and granulocyte colony-stimulating factor (G-CSF), inducing gene expression changes with consequent activation of stemness-associated pathways and methotrexate resistance in lung carcinoma cells [19]. Similarly, exosome Wnt induces resistance to 5-fluorouracil via reprogramming differentiated colorectal cancer cells [63] (Table 2).

## 5. Vesicle-Mediated Resistance to Immunotherapies

Cancers have evolved several strategies to evade immune surveillance; one of these consists of modulating the tumor microenvironment by inhibiting immune response or inducing immune suppressor cells via exosome secretion [154]. Cancer-derived exosomes can impair lymphocyte response, inhibit monocyte differentiation, induce apoptosis in activated T lymphocytes, and downmodulate the cytolytic activity of natural killer (NK) cells [138]. Moreover, vesicles also play a role in immunotherapy resistance. Indeed, these vesicles might act as a decoy target for anticancer immunotherapies [3,155,156]. Vesicles derived from certain tumors can directly bind and neutralize, at least partially, antibody-based drugs. Trastuzumab is a humanized monoclonal antibody that is widely used to treat HER2^+^ breast cancer [157] and targets the extracellular domain of HER2. HER2^+^ breast cancer cells release vesicles expressing this receptor on the surface and competing with that of cancer cells in the binding of trastuzumab, causing a reduction in its bioavailability and efficacy (Table 2) [58]. Moreover, it was recently suggested that a long noncoding RNA named AFAP1-AS1 plays a critical role in establishing trastuzumab resistance [44]. Vesicles mediated the AFAP1-AS1 transfer from trastuzumab-resistant cells to sensitive cells, disseminating drug resistance [44]. The mechanism by which AFAP1-AS1 induces chemoresistance is related to its ability to upregulate HER-2 protein expression through associating with A+U rich RNA binding factor 1 (AUF1) (Table 2) [44]. Rituximab is another antibody used to treat cancer [158] that directly interacts with a vesicle cargo protein.

This chimeric antibody, used in lymphoma therapy, recognizes the cell surface CD20 antigen and induces apoptosis, cytolysis, and complement-dependent cell cytotoxicity (Table 2) [67]. Vesicles isolated from lymphoma specimens and aggressive B-cell lines harbor, at their membrane, a high amount of CD20 that can bind to rituximab, lowering the drug efficacy [67]. The mechanism by which vesicles reduce the efficacy of rituximab is enhanced by the action of ABCA3, as described in Section 2.1 [67].

## 6. Extracellular Vesicles as a Tool to Monitor Chemoresistance

The resistance to anticancer agents remains the leading cause of treatment failure for many oncological patients [3,4,5]. Thus, developing a panel of biomarkers is necessary to identify chemotherapy-resistant patients. As described in the above paragraphs, emerging evidence has revealed the correlation between the onset of chemoresistance and some vesicular components. These data are mainly obtained using vesicles isolated from cancer cell cultures. However, in the last year, a growing number of studies also include the analysis of liquid biopsies from patients to obtain a preliminary in vivo validation [159,160].

In this regard, some examples of lung, prostate, and breast cancers will be described. Janpipatkul et al. analyzed the vesicular miRNA profile of eight patients affected by non-small-cell lung cancer (NSCLS) [161]. The patients were diagnosed with advanced NSCLC with EGFR mutations and started treatment with osimertinib. This drug is a third-generation inhibitor targeting the epidermal growth factor receptor tyrosine kinase (EGFR); it is used to treat a subgroup of NSCLS patients harboring the T790M-EGFR mutation [161]. It is reported that resistance could appear after 9–13 months of treatment [162]. The analysis of vesicular miRNAs isolated from blood samples indicated that miR-323-3p, miR-1468-3p, miR-5189-5p, and miR-6513-5p are suitable candidate biomarkers for the discrimination of osimertinib-resistant from osimertinib-sensitive NSCLC patients [161]. In another study, blood sampling was performed before Pt-based chemotherapy administration and repeated after the occurrence of chemoresistance in 19 NSCLC patients [14]. The analysis of vesicular miRNAs showed that miR-425-3p is abundantly present and might represent a potential biomarker for identifying cisplatin resistance in NSCLC patients [14]. The same miR-425-3p was analyzed in another study that involved 170 patients (76 platinum-resistant and 94 platinum-sensitive). In this case, after collecting serum samples of lung cancer, the authors concluded that miR-425-3p is a good candidate for predicting the clinical response to Pt-based chemotherapy [153]. Furthermore, miR-222-3p significantly correlated with the patient response to chemotherapy; indeed, a study involving 50 patients with lung cancer (NSCLC) demonstrated that high levels of miR-222-3p in serum seem to be predictive of a negative response to gemcitabine treatment [16].

The first-line treatment for prostate cancer, the most commonly diagnosed malignancy in men, is androgen deprivation therapy (ADT); however, about 50% of patients become resistant [163]. In the case of resistance, one of the possible therapeutic interventions is docetaxel, but most patients also acquire docetaxel resistance [164]. A recent study demonstrated that a high copy number of the variant isoform CD44v8-10 mRNA in vesicles is correlated with docetaxel resistance; the study was conducted on blood samples from three groups of patients: controls (n = 15), prostate cancer patients that had not previously received docetaxel therapy (n = 50), and patients with docetaxel-resistant prostate cancer (n = 10). On the contrary, the serum exosomal mRNA of the CD44 standard isoform was not significantly different among the three groups [163]. In the same context, the P-gp level could also serve as a marker for docetaxel resistance in prostate cancer; indeed, in blood exosomes from six docetaxel-resistant patients, the P-gp level was higher than that in patients that had not previously received docetaxel therapy [62]. Another possible strategy to treat ADT-resistant patients is the use of androgen signaling-targeted therapies such as abiraterone and enzalutamide [165]. A study enrolling 36 patients demonstrated that abiraterone and enzalutamide resistance can be predicted by measuring the level of androgen receptor splice variant 7 RNA in plasma-derived vesicles [166]. Moreover, the currently adopted criteria for diagnosing prostate cancer include the use of the prostate-specific antigen (PSA), which showed a low specificity for prostate cancer. This results in an increase in unnecessary biopsies as part of surveillance programs [167]. On the other hand, due to the heterogeneity of primary prostate cancer, tumor biopsy may not necessarily detect the true characteristics of a tumor [168,169]. In light of this, extracellular vesicles collected from body fluids such as urine more likely reflect the current state of cancer cells from which they originated [169]. McKiernan et al. designed and validated a predictive urine-based extracellular vesicle test for prostate cancer using a cohort of 503 men. The amount of exosomal mRNA of prostate cancer antigen 3 (PCA3), ETS-related gene (ERG), and SAM pointed domain-containing Ets transcription factor (SPDEF) was determined for each patient. The results highlight that this test is predictive of high-grade prostate cancer and may contribute to reducing unnecessary biopsies [167,170].

While prostate cancer is the most commonly diagnosed cancer in men, breast cancer is the second leading cause of women’s death [171]. The human epidermal growth factor receptor-2 (HER2) is overexpressed in many breast cancer patients and is the target of the trastuzumab, as described in Section 5. However, a consistent number of patients became resistant to this therapy within 1 year of exposure [172,173,174]. A study showed that vesicular miR-1246 and miR-155 were upregulated in four trastuzumab-resistant patients compared with four patients that responded to therapy [172]. Another study analyzed the expression level of serum vesicular lncRNA-SNHG14 from 38 responding patients and 34 non-responding to trastuzumab therapy, revealing that the lncRNA was upregulated in patients with resistance [43]. It has to be highlighted that trastuzumab is not the only treatment available for breast cancer; indeed, anthracycline and taxane are frequently used in breast cancer patients as neoadjuvant chemotherapies to decrease tumor size and prevent metastasis [175].

However, not all patients respond to the treatment due to intrinsic or acquired resistance [176]. Yang et al. analyzed the expression of the glutathione S-transferase P1 (GSTP1) in exosomes from 30 patients treated with anthracycline/taxane-based neoadjuvant chemotherapy. Results suggested that GSTP1 was significantly higher in the lumen of vesicles from the 14 patients who did not respond to the therapy than in responsive patients [175]. Another study on 131 patients with breast cancer demonstrated that the level of transient receptor potential channel TRPC5 in the membrane of vesicles is a promising candidate as a noninvasive chemoresistance marker [98]. Ning et al. analyzed exosomes isolated from the blood of 93 patients with breast cancer and discovered that the vesicular ubiquitin carboxyl-terminal hydrolase-L1 (UCH-L1) is a useful biomarker for detecting chemoresistance in breast cancer [54].

Similar studies have also been conducted on other cancer types, showing that extracellular vesicles can be used to monitor chemotherapy efficacy [41,42,177,178,179]. In the case of colorectal cancer (CRC), serum samples from patients have been collected and analyzed [180]; in particular, extracellular vesicles containing the lncRNA UCA1 have been identified as responsible for cetuximab resistance phenotype through degradation of miR-204-5p [180]. In the case of ovarian cancer, serum samples were found to contain extracellular vesicles carrying the circular RNA circFoxp1 [177]. This molecule is responsible for cisplatin resistance by targeting miRNAs miR-22 and miR-150-3p [177]. The sorafenib resistance of hepatocellular carcinoma (HCC) has been linked to extracellular vesicles found in the serum of patients containing a lower amount of miR-744 [178]. In the case of renal cell carcinoma (RCC), the resistance to sunitinib treatment is ascribed to an lncRNA, named lncRNA activated in RCC with sunitinib resistance (lncARSR), which binds to miR-34/miR-449 [41]. Glioblastoma is one of the most aggressive tumors, and TMZ is the first-line chemotherapy; however, chemoresistance to TMZ is a common occurrence in glioblastoma and is mediated by miR-1587 and miR-151a contained in serum vesicles [179]. Another study conducted on glioblastoma samples highlighted the miR-4315 released by lymphocytes as responsible for resistance to the anti-PD-1 therapy [42]. In the case of chronic myeloid leukemia (CML), the resistance to imatinib is mediated by miR-365 released in extracellular vesicles [181]. In all the mentioned studies, the extracellular vesicles produced by resistant cancer cells can induce the resistant phenotype in sensitive recipient cells [41,42,177,178,179]. Therefore, circulating serum vesicles can be considered a promising tool for identifying the cancer stage and responsiveness to treatment.

## 7. Conclusions

Extracellular vesicles represent a highly attractive source of biomarkers because these vesicles can be easily collected from different body fluids by liquid biopsy. This is greatly relevant in terms of the life quality of cancer patients because it is possible to monitor cancer treatment response via a noninvasive procedure. Compared to other types of molecules that can be isolated from body fluids, such as circulating miRNA or circulating tumor DNA, extracellular vesicles are highly preferable because their membrane protects cargo(s) from degradation (Figure 1). Moreover, vesicle analysis offers the opportunity to isolate multiple biomarkers simultaneously. Indeed, comparing a panel of markers may give more accurate results or information to predict therapeutic response.

However, still, some limitations exist: an aspect to consider is the type of body fluid used for collecting extracellular vesicles. Urine is a suitable source from which to isolate vesicles for detecting biomarkers of the urinary system such as those signifying prostate, bladder, and kidney cancers. Another body fluid that is used in the detection of biomarkers for detecting oral and lung cancer is saliva [182]. Saliva is the most proximal body fluid in oral cancer and is easily accessible in a noninvasive manner [183]. An example of the potential use of saliva as a source of biomarkers to predict oral cancer is reported by Gai et al. In this study, miR-302b-3p and miR-517b-3p were found selectively enriched in salivary EVs from 16 oral squamous cell carcinoma patients compared to those from 6 healthy controls [184]. Moreover, He et al. showed that the level of miR-24-3p from the salivary vesicles of 45 OSCC patients is significantly higher compared to that of 10 normal controls [185]. Another example is represented by the salivary vesicle GOLM1-NAA35 chimeric RNA, which was proposed as a noninvasive biomarker candidate for reliable assessment of therapeutic response, recurrence, and early detection of esophageal squamous cell carcinoma [186].

However, for other cancer types, blood is the source of choice for vesicle isolation. In this respect, it has to be stressed that blood contains a large number of proteins that can contaminate exosome preparation, making the detection of poorly expressed biomarkers difficult.

Other prerequisites for developing a functional clinical test are cheapness and easy execution. Currently, the exosome isolation methods are time-consuming or expensive. Nevertheless, the feasibility of using extracellular vesicles biomarkers in precision medicine is demonstrated by the fact that in 2019, the EPI test, the first EV-based Clinical Laboratory Improvement Amendments (CLIA)-validated and clinically available test, received the FDA breakthrough design designation for fast-tracked approval process. It is a noninvasive urine exosome-based diagnostic test that measures the RNA of prostate cancer antigen 3 (PCA3), ETS-related gene (ERG), and SAM pointed domain-containing Ets transcription factor (SPDEF). This test can guide physicians in determining the need for a prostate biopsy in patients 50 years of age or older with a prostate-specific antigen (PSA) in the grey zone [187].

Moreover, as extracellular vesicles naturally carry bioactive molecules between cells, some studies suggest that these may serve to deliver drugs or RNAs in the context of cancer therapy or to reverse drug resistance [140,188]. Indeed, extracellular vesicles may offer some advantages as delivery carriers, such as biocompatibility, non-cytotoxicity, and low immunogenicity [140,189,190]. In this light, Liang et al. showed that the administration of engineered extracellular vesicles loaded with the 5-fluorouracil and the miR-21 inhibitor oligonucleotide (miR-21i) to resistant colon cancer cells effectively reverses drug resistance [188,191]. Therefore, even if the way is still long, all the mentioned findings strongly support the development of a therapeutic and diagnostic era based on exosomes.

## Figures and Tables

**Table 1:** Chemoresistance associated with extracellular vesicles.

EV Cargo Type	Cancer Type	Drug Resistance	Ref.
miRNA	Lung cancer	Cisplatin	[14,15]
		Gemcitabine	[16]
		Gefitinib	[17,18]
		Methotrexate	[3,19]
	Gastric cancer	Paclitaxel	[20]
		Cisplatin	[20]
		Doxorubicin	[21]
	Breast cancer	Paclitaxel	[22]
		Doxorubicin	[22]
		Docetaxel	[23,24]
		Gemcitabine	[24]
		CDK4/6 inhibitor	[25]
		Epirubicin	[24]
	Head and neck cancer	Cisplatin	[26,27]
	Pancreatic cancer	Gemcitabine	[28]
	Hepatic carcinoma	5-Fluorouracil	[29]
		Oxaliplatin	
		Gemcitabine	
		Sorafenib	
	Colorectal cancer	5-Fluorouracil	[30,31]
		Oxaliplatin	[31]
	Ovarian cancer	Cisplatin	[32]
		Paclitaxel	[33,34]
	Lymphoma	Gemcitabine	[35]
	Prostate cancer	Cisplatin	[36]
		Doxorubicin	
		Docetaxel	
lncRNA	Lung cancer	Gefitinib	[37]
	Ovarian cancer	Cisplatin	[38]
	Esophageal cancer	Gefitinib	[39]
		Sorafenib	[40]
		Camptothecin	[40]
		Doxorubicin	[40]
	Renal cell carcinoma	Sunitinib	[41]
	Glioblastoma	Temozolomide	[42]
	Breast cancer	Trastuzumab	[43,44]
	Colorectal cancer	Oxaliplatin	[45,46]
	Bladder cancer	Cisplatin	[47]
circRNA	Colorectal cancer	Oxaliplatin	[48]
	Lung cancer	Cisplatin	[49]
	Gastric cancer	Cisplatin	[50]
mRNA	Glioma	Temozolomide	[51]
	Lung cancer	Cisplatin	[52]
		Gemcitabine	[52]
	Osteosarcoma	Doxorubicin	[53]
Protein	Breast cancer	Adriamycin	[54]
		Docetaxel	[55]
		Doxorubicin	[56]
		Paclitaxel	[57]
		Trastuzumab	[58]
	Ovarian cancer	Cisplatin	[59]
		Paclitaxel	[60]
		Platinum-based therapy	[60]
		Cisplatin	[61]
	Osteosarcoma	Doxorubicin	[53]
	Prostate cancer	Docetaxel	[62]
	Colorectal cancer	5-Fluorouracil	[63,64]
	Pancreatic cancer	Gemcitabine	[65]
	B-cell lymphoma	R-CHOP	[66]
		Rituximab	[67]
	Glioblastoma	Temozolomide	[68]

**Table 2:** Exosomal bioactive molecules involved in the induction of drug resistance.

Section	Effectors	Mechanisms	Cancer Type	Drug Resistance	Marker	Ref.
2	Acidic environment	Increased EV secretion	Melanoma	Cisplatin	Rab-5b	[78]
					CD63	
	ABCA3 depletion	Decreased EV secretion ameliorates drug sensitiveness	B-cell lymphomas	Doxorubicin Pixantrone	CD9	[79]
					CD63	
					ADAM10	
	Annexin A3	Increased EV secretion	Ovarian cancer	Pt-drugs	TEM	[81]
					Hsp70	
	P-gp	Delivering through EVs	Prostate cancer	Docetaxel	CD9	[62]
				Doxorubicin		
	MDR-1 mRNA/P-gp	Delivering through EVs	Osteosarcoma	Doxorubicin	CD63	[53]
	P-gp	Delivering through EVs	Breast cancer	Docetaxel	TSG101	[55]
	TrpC5	Induction of P-gp expression	Breast cancer	Adriamycin	Flotillin-2	[56]
	TrpC5	Delivering through EVs	Breast cancer	Anthracycline Taxane	CD63	[98]
					Flotillin-1	
	UCH-L1	Induction of P-gp expression	Breast cancer	Adriamycin	CD63	[54]
					Flotillin-1	
	LINC00355	Induction of P-gp expression	Bladder cancer	Cisplatin	CD9	[47]
					CD63	
	miR-1246	Downregulation of caveolin1,	Ovarian cancer	Paclitaxel	CD63	[34]
		upregulation of P-gp				
	circ_PIP5K1A	Downregulation of	Lung cancer	Cisplatin	CD81	[49]
		miR-101			CD63	
	linc-VLDLR	Upregulation of ABCG2	Hepatocellular cancer	Sorafenib, camptothecin	NTA	[99]
				Doxorubicin		
	ABCA3	Increased exosome secretions	Malignant lymphoma	Rituximab	Flotillin-2	[67]
					Alix	
					CD9	
					CD63	
	ATP7A	Increased Pt-drug excretion	Ovarian cancer	Pt-based drugs	Microscopy of labeled vesicles	[61]
	ATP7B					
	miR-4717-5p	Downregulating of ENT2	Lymphoma	Gemcitabine	CD9	[35]
				Cytarabine	CD63	
	miR-1236	SLC9A1 downregulation ameliorates drug sensitiveness	Breast cancer	Cisplatin	CD9	[100]
					CD63	
					CD81	
					HSP70	
	Sulfasalazine (SAS)	EVs from cells treated	Melanoma	Immune checkpoint blockade (ICB) therapy	TEM	[101]
		with xCT inhibitor SAS reduced ICB therapy efficacy				
3	miR-145/−34a	Lower apoptosis level	Colon cancer	5-Fluorouracil	NTA	[30]
	Bone marrow stromal cell derived EVs	Increase in antiapoptotic proteins	Multiple myeloma	Bortezomib	Hsp90	[102]
					Hsp70	
					CD63	
					Flotillin-1	
	SNHG14	Modulation of Bcl-2/Bax	Breast cancer	Trastuzumab	CD9	[43]
					CD63	
					CD81	
					Alix	
	PART1	Modulation of miR-129	Esophageal squamous cell carcinoma	Gefitinib	CD63	[39]
					CD81	
	miR-21	Modulation of PTEN/PDCD4	Squamous cell carcinoma	Cisplatin	CD81	[27]
					CD68	
	miR-32-5p	PI3K/Akt pathway activation	Hepatocellular carcinoma	Multidrug	CD63	[29]
				resistance	TSG-101	
					Flotillin-1	
	miR-27a	TP53	Prostate cancer	Cisplatin, docetaxel, doxorubicin	CD63	[36]
					CD9	
	EVs from HBV-associated	Chaperone-mediated autophagy	Liver cancer	Oxaliplatin	CD63	[103]
	liver cancer cells				CD9	
	miR-425-3p	Modulation of AKT1	Lung cancer	Cisplatin	TEM	[14]
					NTA	
	PD-L1	Activation of	Glioblastoma	Temozolomide	TSG101	[68]
		AMPK/ULK1				
		pathway				
	circ-PVT1	Modulating	Gastric cancer	Cisplatin	CD63	[50]
		miR-30a-5p			CD9	
	SBF2-AS1	Modulation of miR-151a	Glioblastoma	Temozolomide	CD63	[42]
					CD81	
	MGMT	Delivering through EVs	Glioma	Temozolomide	CD63	[51]
					CD81	
4	miR-210	Inhibition of apoptosis	Pancreatic cancer	Gemcitabine	CD63	[104]
					CD81	
					GM130	
	miR-92a-3p	FBXW7, MOAP1	Colorectal cancer	5-Fluorouracil	CD63	[31]
				Oxaliplatin	CD81	
					TSG101	
	G-CSF, IL-6	Gene expression changes	Lung carcinoma	Methotrexate	EV communication in	[19]
	Activin-A				cocultured cells was blocked by xyloside	
	Wnt	Reprogramming of differentiated cancer cells	Colorectal cancer	5-Fluorouracil	CD81	[63]
5	HER2	Antibody binding	Breast cancers	Trastuzumab	CD63	[58]
					Flotillin-1	
	AFAP1-AS1	Upregulation HER-2	Breast cancers	Trastuzumab	TSG101	[44]
					CD81	

## Data Availability

Not applicable.
